# Bi_2_Te_3_-Based Thermoelectric Films Fabricated by Magnetron Sputtering

**DOI:** 10.3390/ma19102111

**Published:** 2026-05-17

**Authors:** Weiye Geng, Yongcheng Du, Size Lou, Hao Sun, Peng’an Zong

**Affiliations:** College of Materials Science and Engineering, Nanjing Tech University, Nanjing 210009, China

**Keywords:** Bi_2_Te_3_ thin films, magnetron sputtering, thermoelectric performance, process parameters, post-treatment and doping

## Abstract

Bi_2_Te_3_-based materials are benchmark room-temperature thermoelectrics, widely used in refrigeration, waste heat recovery, and microdevice thermal management. Magnetron sputtering demonstrates significant potential as an effective strategy for the mass production of superior Bi_2_Te_3_ thin films, offering advantages such as dense microstructure, controllable composition, good repeatability, and compatibility with semiconductor processes. However, existing studies largely focus on individual factors affecting film properties, lacking a systematic understanding of the interrelationships among process parameters, microstructures, and thermoelectric performance. Poor comparability across studies due to varying deposition conditions further limits insight into key controlling mechanisms. Recent efforts have centered on three regulatory aspects in magnetron sputtering: (1) optimization of sputtering parameters (e.g., power, pressure, temperature, and target composition); (2) post-annealing treatment; and (3) doping modification. Notable progress has been made in enhancing thermoelectric performance through these approaches. This paper provides a comprehensive overview of recent advancements in the fabrication of Bi_2_Te_3_ thin films via magnetron sputtering, focusing on how process parameters, post-treatment, and doping affect microstructure, stoichiometry, and thermoelectric properties. The aim is to elucidate structure-performance correlations and guide the optimized preparation of high-performance films.

## 1. Introduction

**Figure 1:**
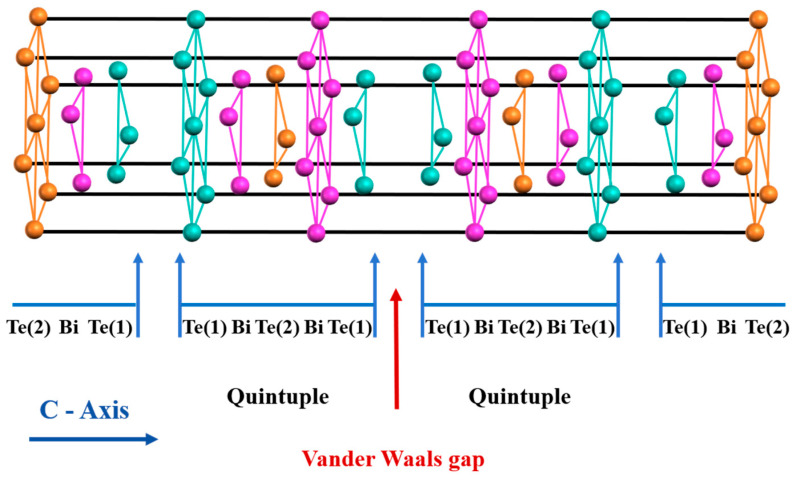
Schematic diagram of the layered structure and bonding characteristics of Bi_2_Te_3_ crystal along the c-axis. Note: Red atoms are Bi atoms. Cyan atoms are Te(1) atoms, which are bonded to each other via van der Waals forces in adjacent five-layer structures. Orange atoms are Te(2) atoms, which are covalently bonded to Bi.

At present, Bi_2_Te_3_-based compounds and their solid solutions represent the premier thermoelectric materials operating around room temperature, playing an essential role in both solid-state cooling and waste heat harvesting technologies [1,2]. At ambient conditions, the *V*–*VI* semiconductor Bi_2_Te_3_ crystallizes in a rhombohedral lattice (space group R$\bar{3}$m). As depicted in Figure 1, its structural framework is defined by the c-axis stacking of Te(1)-Bi-Te(2)-Bi-Te(1) quintuple layers (QLs), which are connected exclusively through weak van der Waals interactions, and this unique crystal structure leads to significant anisotropy in its thermoelectric transport properties [3,4]. Bi_2_Te_3_ is a narrow bandgap semiconductor with a bandgap of ~0.145 eV and carrier concentration between 10^18^ and 10^19^ cm^−3^. Through doping with elements such as Se or halogens to achieve n-type behavior, alongside Sb for p-type doping, ensuring the successful synthesis of robust thermoelectric compounds [5].

The thermoelectric effect refers to the physical mechanisms through which a material generates an electrical potential difference under a temperature gradient (Seebeck effect) or undergoes heat absorption or dissipation driven by an electric current (Peltier effect) [6]. The conversion efficiency of thermoelectric compounds is generally evaluated using the efficiency of thermoelectric materials is evaluated by the dimensionless parameter *ZT* = *S*^2^*σ*T/*к*. In this expression, *T* denotes the absolute temperature, *S* represents the Seebeck coefficient, and *σ* stands for the electrical conductivity. Furthermore, the overall thermal conductivity *к* encompasses both the lattice (*к_L_*) and electronic (*к_e_*) components [7]. Achieving a superior *ZT* requires maintaining superior electrical transport performance (evaluated by the power factor *PF* = *S*^2^*σ*) whilst concurrently suppressing the thermal conductivity. Nevertheless, decoupling and individually maximizing these variables remains a substantial obstacle due to the strong interrelation among *S*, *σ*, and *к_e_* [8]. Bi_2_Te_3_-based systems achieving an approximate *ZT* of 1.0 in the vicinity of room temperature, remain the primary choice for commercial thermoelectric refrigeration devices. They are extensively utilized in applications such as infrared detectors, laser diode cooling, automotive refrigerators, and precision temperature control systems [9,10].

With the rapid development of microelectronic devices and wearable electronics toward miniaturization, integration, and low power consumption, conventional bulk thermoelectric materials are struggling to meet the demands of precise micro-zone temperature control and energy harvesting [11]. To address this challenge, thin-film thermoelectrics have emerged as a key research direction. First, thin films possess a higher specific surface area and shorter heat transfer distance, facilitating fast thermal response and efficient thermal management. Second, the thin-film geometry is compatible with microelectromechanical systems (MEMS), enabling the fabrication of miniature thermoelectric devices for on-chip cooling, microsensors, and wearable electronics [12,13]. Third, the interfaces and nanostructures within thin films can introduce strong phonon scattering, effectively reducing lattice thermal conductivity and offering the potential for achieving higher *ZT* values [14]. Furthermore, the thin-film platform provides an ideal system for investigating quantum confinement effects and topological electronic states in low-dimensional systems—as a prototypical three-dimensional topological insulator, Bi_2_Te_3_ in thin-film form exhibits significant potential for applications in spintronics and quantum computing [15].

However, compared with bulk materials, the preparation of thin films faces additional challenges: incompatibilities in lattice constants and coefficients of thermal expansion (CTE) across the film-substrate interface intrinsically trigger internal stress and defect formation, and adhesion issues; the nonequilibrium deposition conditions during thin-film growth may readily cause compositional deviations from stoichiometry; and controlling compositional uniformity and structural consistency along the film thickness direction becomes more difficult [16]. Therefore, developing reliable thin-film fabrication techniques and gaining a deep understanding of their growth mechanisms are essential for advancing the practical applications of thermoelectric thin-film materials. Numerous techniques have been established for fabricating Bi_2_Te_3_ thin films, primarily involving MBE (molecular beam epitaxy) and CVD (chemical vapor deposition), electrochemical deposition (ECD), pulsed laser deposition (PLD), and magnetron sputtering (MS) [17,18,19,20,21]. MBE can produce epitaxial thin films with atomic-level precision; however, the high cost of equipment and low deposition rate hinder its large-scale application. CVD enables the fabrication of thin films with complex morphologies, but issues such as precursor toxicity, process reproducibility, and substrate temperature limitations remain prominent. Electrochemical deposition offers simple processing and low cost, yet the resulting films often suffer from insufficient density and adhesion, and the process requires conductive substrates. PLD ensures good consistency in composition transfer, but the deposition uniformity is limited, making large-area preparation difficult. Magnetron sputtering, a physical vapor deposition technique, has attracted significant interest within the realm of thermoelectric film deposition, owing to its distinct benefits [22]. By applying an orthogonal electromagnetic field on the target surface, magnetron sputtering enhances the plasma density of the glow discharge, significantly increasing the sputtering rate and enabling the deposition of premium thin layers under mild thermal conditions [23]. This method offers several advantages, including dense film formation with strong adhesion to the substrate, good controllability of composition, ease of scaling up for mass production, high process reproducibility, alongside its exceptional versatility in material deposition, rendering it highly ideal for engineering multilayered films and superlattice configurations [24,25]. Based on the above benefits, Among various deposition technologies, magnetron sputtering has proven to be a highly industrially promising technical routes for the preparation of Bi_2_Te_3_ thermoelectric thin films.

Recently, substantial advancements have been achieved in the research on Bi_2_Te_3_ thin films prepared by magnetron sputtering, However, existing work have mostly focused on the effects of doping modification, annealing, and other processes on material performance, while a systematic synthesis of the intrinsic correlations among “process parameters—microstructure—thermoelectric properties” is still lacking [26,27]. Owing to differences in deposition conditions across studies, the reported film structures and properties are often poorly comparable, which hinders a deeper understanding of the key controlling factors. Therefore, a comprehensive and systematic review of the research progress on Bi_2_Te_3_ magnetron-sputtered thin films sputtering is urgently needed.

This review aims to systematically summarize the current status of Bi_2_Te_3_ thin films prepared by magnetron sputtering, with an emphasis on analyzing the influence of key processing parameters—such as sputtering power, working pressure, substrate thermal conditions, target composition, and substrate type—as well as post-treatment methods including annealing and multilayer structures, and doping with elements such as Se, Sb, and Mn, regarding the thermoelectric behavior, chemical makeup, and structural evolution of the fabricated films. By clarifying the intrinsic principles governing each processing factor, this work reveals the intrinsic relationships between structural regulation and performance optimization, summarizes the breakthrough advances achieved so far, and identifies the main challenges and future research directions. Ultimately, this article aims to offer a comprehensive roadmap for scientists in this field, specifically those focusing on Bi_2_Te_3_ thin-film materials and related thermoelectric devices., thereby promoting further development and application of magnetron sputtering technology in this field.

## 2. Fundamentals of Magnetron Sputtering Technology

### 2.1. Basic Principles of Magnetron Sputtering

As a widely utilized physical vapor deposition (PVD) method, magnetron sputtering operates on the basis of glow discharge. Fundamentally, this procedure utilizes an electric field inside a vacuum environment to ionize a precursor gas—most commonly argon—thereby generating a plasma. Subsequently, the applied electric field accelerates these newly formed positive ions, driving them to forcefully strike the target surface. Through momentum transfer, target atoms are ejected (sputtered) and subsequently accumulate on the substrate surface, ultimately yielding a thin layer. Equally important, the impact of energetic ions also causes electron ejection from the target; these secondary electrons are accelerated by the electric field and collide with neutral gas atoms to sustain the self-sustaining glow discharge [28,29].

The key to magnetron sputtering lies in introducing orthogonal electromagnetic fields above the target surface. By placing permanent magnets or electromagnetic coils behind the target, a crossed electric and magnetic field configuration is implemented. Electrons in the crossed electromagnetic fields experience a Lorentz force, which confines their trajectories to the vicinity of the target surface, causing them to move in helical paths. This greatly extends the path length and residence time of electrons within the plasma region [30]. Dramatically enhances the collision frequency between the confined electrons and working gas atoms, allowing a high-density plasma to be maintained at relatively low working pressures and low voltages, thereby enhancing the sputtering rate and deposition efficiency. Sputtering yield is an important parameter describing sputtering efficiency, defined as the number of atoms ejected from the target surface per incident ion. Variables including the kinetic energy, atomic mass, and trajectory of the bombarding ions, coupled with the inherent composition and crystallographic structure of the source material, collectively dictate the overall sputtering yield [31].

### 2.2. Sputtering Modes and System Configurations

Depending on the power supply type and operating mode, various magnetron sputtering configurations exist, with direct current (DC) and radio frequency (RF) and co-sputtering being the most prevalent. Different modes are suitable for different target material characteristics and preparation requirements. DC sputtering is commonly used for conductive targets due to its high deposition rate and simple operation. RF sputtering, typically operated at 13.56 MHz, is preferred for insulating or poorly conductive targets, as it prevents charge accumulation on the target surface. Co-sputtering, which employs two or more targets simultaneously, enables the deposition of composite or alloy films with tunable compositions. In addition to these conventional modes, advanced configurations such as closed-field unbalanced magnetron sputtering (CFUBMS) and pulsed magnetron sputtering (PMS) have been developed to further improve film density and process stability [32]. Using PMS, Wojciechowski et al. successfully deposited Bi_2_Te_3_ thermoelectric thin films with competitive power factors [33].

#### 2.2.1. DC Magnetron Sputtering

Direct current (DC) sputtering represents the most fundamental and widely adopted configuration, specifically tailored for metallic source materials with good electrical conductivity and some semiconductor targets. The formation of a persistent glow discharge is driven by the application of a DC bias between the cathodic target and the anodic substrate. The advantages of this method include simple equipment, high deposition rate, and good process stability [34]. For semiconductor materials such as Bi_2_Te_3_, the conductivity of the target must be considered when using DC sputtering. Typically, high-purity (>99.99%) hot-pressed or melted targets are used, whose resistivity is sufficiently low to maintain a stable discharge.

#### 2.2.2. RF Magnetron Sputtering

RF sputtering is suitable for depositing insulating targets or materials with poor electrical conductivity. The RF power supply (typically the internationally standardized frequency of 13.56 MHz) delivers energy to the plasma through capacitive coupling. Due to the vast disparity in mass and resultant velocity between electrons and heavy ions, a spontaneous negative DC bias develops at the target boundary, which causes positive ions to accelerate toward and bombard the target during the negative half-cycle of the RF period. The advantages of RF sputtering include the ability to directly sputter insulating targets, as well as high ionization efficiency and plasma density. However, its disadvantages are a complex impedance matching system, higher equipment costs, and typically lower deposition rates compared to DC magnetron sputtering [35]. For the preparation of Bi_2_Te_3_ thin films, RF sputtering is often employed for precise composition compensation in multi-target co-sputtering systems, or when the target conductivity is insufficient to maintain a stable DC discharge due to the semiconducting nature of the material [36].

#### 2.2.3. Co-Sputtering

Co-sputtering refers to the simultaneous use of two or more sputtering sources, allowing the film stoichiometry to be adjusted by separately controlling the deposition flux (or sputtering power) from each target. This mode offers unique advantages for preparing doped films or composition-gradient films. For example, co-sputtering using a Bi_2_Te_3_ target and Co target can produce Bi_2_Te_3_-Co films with varying Co content [37]. The advantage of co-sputtering is its flexibility in composition tuning; however, challenges to be addressed include matching the deposition rates of different targets and ensuring composition uniformity over large-area deposition.

#### 2.2.4. Bias Sputtering

Substrate bias sputtering is an important means of optimizing thin film quality. The imposition of a modest negative potential (commonly between −50 and −200 V) on the substrate facilitates the extraction of positive ions from the plasma, directing them to strike the advancing film surface, producing an “ion assistance” effect. Such ion bombardment can enhance surface atom mobility, increase film density, and modify the texture orientation of the film. However, excessively high bias voltage may lead to resputtering or introduce damage-related defects [38].

### 2.3. Growth Morphology and Texture of Thin Film Bismuth Telluride-Based Materials

After sputtered atoms arrive at the substrate surface, they first bind to the substrate through physical or chemical adsorption. Adatoms possess high mobility on the substrate surface, allowing them to diffuse and collide with each other to form atomic clusters. When the cluster size exceeds the critical nucleation size, stable nuclei are formed [39]. Depending on the interactions among the substrate, the film material, and the sputtering parameters, thin-film nucleation and subsequent development are generally categorized into three distinct regimes based on their morphological characteristics [40].

#### 2.3.1. Island-like Morphology (015 Preferred Orientation)

**Figure 2:**
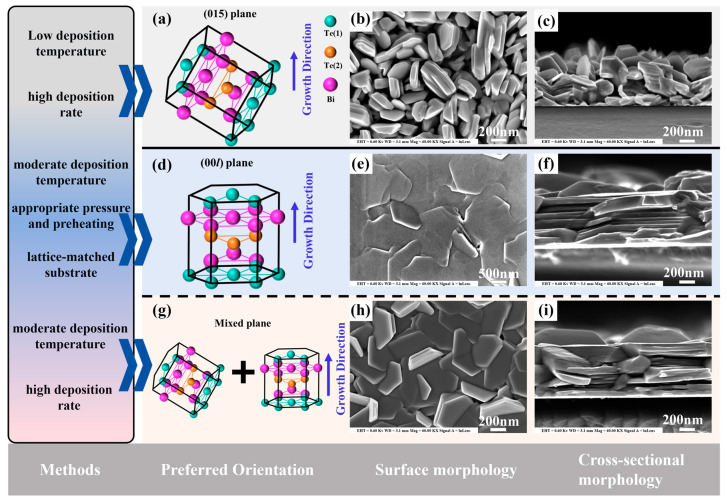
(**a**) Orientation of Bi_2_Te_3_ grains with (015) texture. (**b**) Surface morphology image of island growth. (**c**) Cross-sectional morphology image of island growth. (**d**) Orientation of Bi_2_Te_3_ grains with (00*l*) texture. (**e**) Surface morphology image of layered growth. (**f**) Cross-sectional morphology image of layered growth. (**g**) Mixed orientation of Bi_2_Te_3_ grains with both the (00*l*) and (015) directions. (**h**) Surface morphology image of mixed growth. (**i**) Cross-sectional morphology image of mixed growth. Note 1: Since the tellurium atoms reside in two distinctly different chemical environments, the Te-Bi bonds predominantly exhibit covalent characteristics, whereas the adjacent quintuple layers are held together by much weaker van der Waals forces between the Te(1)-Te(1) atomic layers. To accurately reflect this spatial asymmetry and bonding difference in the structural diagram, Te(1) and Te(2) are represented with different colors Note 2: The SEM images showing the typical microstructures are adapted from Ref [28].

During the preparation of bismuth telluride (Bi_2_Te_3_)-based thin films by magnetron sputtering, an island-like morphology is primarily observed under conditions of limited surface atomic diffusion, such as low deposition temperatures or high deposition rates. Under these conditions, the film preferentially nucleates in the form of discrete nuclei and subsequently undergoes island growth and coalescence. Tao et al. directly observed this evolutionary pathway through AFM/SEM characterization of Bi_2_Te_3_/PET room-temperature DC magnetron-sputtered thin films: small islands formed at the initial stage increased in height and came into contact with one another as the film thickness increased, eventually coalescing into larger grains [41]. This process is accompanied by a dynamic evolution of surface roughness, providing direct evidence for the island growth mechanism under low-temperature sputtering conditions. While the pronounced (015) preferred orientation observed in XRD patterns is not directly equivalent to island growth, both the (015) texture and the granular morphology reflect similar non-equilibrium kinetic conditions. In terms of thermoelectric performance, phonons undergo intense scattering at the numerous nanostructured interfaces created during island-like film evolution, which significantly hampers their propagation. Through the strategic restriction of the phonon mean free path, the lattice-governed heat transport is effectively driven beneath its intrinsic bulk threshold. Concurrently, the potential barriers established at grain boundaries exert an ‘energy filtering’ influence; by obstructing the transport of cold carriers, these barriers elevate the mean energy of the remaining charge population, leading to a marked boost in the film’s Seebeck coefficient. The study by Chen et al. confirmed that this (015)-textured structure exhibits excellent power factor potential even in the early, non-continuous stage of film formation [40]. Furthermore, as shown in Figure 2a–c, the granular microstructure resulting from island growth can effectively relax internal stresses caused by thermal expansion mismatch or bending, thereby improving the adhesion and cyclic bending lifetime of the thin film on flexible substrates.

#### 2.3.2. Lamellar Morphology (00*l* Preferred Orientation)

Throughout the synthesis of Bi_2_Te_3_-based films employing magnetron sputtering techniques, the formation of layered growth essentially depends on whether the in-plane migration ability of atoms after arriving at the substrate surface is sufficient to support preferential rearrangement along the basal plane. Bi_2_Te_3_ itself is stacked along the c-axis in quintuple layers, with the cleavage plane perpendicular to the c-axis. When (00*l*) diffraction peaks dominate the XRD pattern, it indicates that the *c*-axis of the film is essentially perpendicular to the substrate, as illustrated in Figure 2d–f, a horizontal alignment of the layered planes relative to the substrate is observed. This observation serves as a clear diffraction evidence for a strongly lamellar (00*l*) texture. Furthermore, the transport of heat and charge within the (00*l*) basal planes of Bi_2_Te_3_ is markedly superior to that occurring across the c-axis; therefore, (00*l*) texture is generally regarded as an important structural signature of layered preferential growth [42].

From the perspective of process parameters, the substrate temperature primarily determines the surface diffusion capability. Kim et al. found in co-sputtered Bi_2_Te_3_ thin films that as the deposition temperature increased, clear hexagonal grains appeared on the film surface above 290 °C, indicating that higher atomic mobility favors crystal rearrangement along low-surface-energy orientations [43]. However, excessively high temperatures can cause compositional deviation and induce phase transitions. Thus, the effect of temperature on layered growth exhibits a dual nature: promoting rearrangement at moderate temperatures and causing instability at excessive temperatures. The working pressure influences the layered orientation by controlling the collisional scattering of particles in Ar and their kinetic energy upon reaching the substrate. Nakano et al. pointed out that Higher argon pressure leads to a wider initial track width in the magnetron sputtering configuration, thereby affecting particle scattering and the sputtering rate [44]. Kianwimol et al. further showed that under appropriate pressure conditions combined with substrate pre-heating, flexible Bi_2_Te_3_ thin films can simultaneously achieve near-stoichiometric composition and strong (00*l*) orientation [45]. Their work explicitly identified the formation of (00*l*) orientation and subsequent diffusion as the key to obtaining this texture. The sputtering power/deposition rate indirectly affects the layered growth window by modifying the particle flux arriving at the substrate, surface residence time, and the resulting composition and grain size. The substrate type is also important. Shang et al. obtained clearly (00*l*)-textured Bi_2_Te_3_ thin layers synthesized via co-sputtering on single-crystal MgO substrates maintained at 400 °C, demonstrating that substrates with a template effect or better interfacial matching more readily induce layered preferential alignment [46].

#### 2.3.3. Mixed Granular-Lamellar Morphology

During the preparation of bismuth telluride-based thin films by magnetron sputtering, a mixed granular-lamellar morphology is sometimes observed, which superficially resembles a Stranski-Krastanov (SK)-type “layer-then-island” process, though it is more accurately regarded as a mixed texture without direct in situ evidence of the nucleation pathway. As shown in Figure 2g–i: in this mode, a continuous or semi-continuous wetting layer initially forms on the substrate. Subsequently, due to interfacial energy, strain accumulation, and a renewed imbalance between surface diffusion and deposition flux, three-dimensional island-like grains nucleate and continue to grow. Zhu et al. observed a texture transformation in Bi_0.5_Sb_1.5_Te_3_ thin films deposited by DC magnetron sputtering [47], which they discussed in the context of the SK model; this transformation is essentially a thermally driven recrystallization of the as-deposited (015) texture into a (00*l*) texture. The minimization of cumulative interfacial energy acts as the primary catalyst for this structural evolution, reflecting a fundamental thermodynamic equilibrium process, the intensification of the layered texture is primarily driven by the in-plane transport and impingement of adatoms during the growth process. From the perspective of process parameters, the substrate temperature primarily determines the direction of this “layer vs. island” competition. When the temperature is too low, surface atomic migration is insufficient, and the growth approaches pure island nucleation. The working pressure influences the balance between “initial spreading” and “island stacking” by modulating the collisional scattering of sputtered particles in the Ar atmosphere and their kinetic energy upon reaching the substrate. Under appropriate working pressure combined with substrate pre-heating, nearly stoichiometric and highly (00*l*)-oriented Bi_2_Te_3_ thin layers can be successfully synthesized. The underlying mechanism is related to the formation of the (00*l*) orientation and subsequent diffusion processes.

## 3. Effects of Process Parameters, Post-Treatment, and Doping Modification on the Structure and Properties of Bi_2_Te_3_ Thin Films

During the highly non-equilibrium kinetic deposition process of magnetron sputtering, entropy (*S*) is not merely a passive physical parameter, but a critical factor determining the final microstructure, composition, and orientation of the thin films. By minimizing the system’s Gibbs free energy (*G* = *H* − *TS*, where *H* is enthalpy, *T* is absolute temperature, and *S* is entropy), entropy exerts a decisive influence on the ultimate physical morphology of Bi_2_Te_3_ thin films across three core dimensions:

First, although high sputtering rates can trap atoms in kinetic minimums, resulting in rough polycrystalline or amorphous states, an entropy increase—given sufficient thermal activation—drives surface adatoms to overcome kinetic migration barriers and rearrange into a (00*l*)-oriented layered structure characterized by the lowest surface free energy. The work of Tang et al.explicitly points out that, under stable growth kinetics and sufficient thermodynamic (entropic) driving forces, the films can overcome random stacking and exhibit a highly ordered, layer-by-layer growth mode [48]. This demonstrates that entropy significantly influences the driving forces governing the morphological and orientational evolution of magnetron-sputtered Bi_2_Te_3_ films.

Second, the existence of configurational entropy dictates that an absolutely perfect crystal lattice is unstable at finite temperatures. To maximize configurational entropy and thereby lower the overall free energy, the spontaneous formation of a certain concentration of tellurium vacancies (V_Te_) and antisite defects (Bi_Te_ or Te_Bi_) within the Bi_2_Te_3_ lattice is a thermodynamic inevitability. As confirmed by A. Hashibon et al. through first-principles density functional theory (DFT) calculations, such intrinsic point defects possess extremely low formation energies in Bi_2_Te_3_ [49]. The compensation effect of configurational entropy allows these defects to form abundantly at room temperature and typical deposition temperatures, thereby directly locking in the semiconductor type and electrical characteristics of the films during the initial deposition stages.

Finally, entropy is also a decisive factor in phase transitions and compositional deviations, as the high-entropy nature of the gas phase governs the phase stability of the films upon heating. Experiments conducted by K. Singkaselit et al. demonstrate that when the annealing temperature reaches 350 °C and above, the immense “vaporization entropy increase”—induced by Te atoms escaping the solid-state lattice into the gas phase—not only reduces the internal film density and increases surface roughness, but also directly triggers an irreversible structural collapse [50]. This ultimately drives the phase transition from Bi_2_Te_3_ to a Bi-rich BiTe structure.

### 3.1. Influence of Sputtering Process Parameters

During the preparation of Bi_2_Te_3_-based thin films by magnetron sputtering, the selection of deposition parameters directly determines the microstructure, stoichiometry, and ultimately the thermoelectric properties of the films. By precisely tuning the energy distribution and mass transport throughout the magnetron-based plasma deposition cycle, effective control over the quality of thin film growth can be achieved.

#### 3.1.1. Sputtering Power and Deposition Rate

Sputtering power is a core physical parameter that controls the deposition rate, while the initial kinetic energy of adatoms is primarily governed by the target voltage (i.e., the discharge voltage), which is coupled to the sputtering power under typical operating conditions. Increasing the sputtering power significantly enhances the intensity of ion bombardment on the target surface and raises the instantaneous flux of ejected particles, thereby promoting grain growth while improving the deposition efficiency. Studies have shown that higher sputtering power endows the deposited atoms with greater initial kinetic energy. Particularly in DC magnetron sputtering, the direct transfer of kinetic energy results in significantly higher surface mobility of adatoms compared to RF sputtering at the same power level. This increased mobility facilitates the diffusion of atoms across the surface, allowing them to settle into the lowest-energy crystallographic positions, thereby improving film density, adhesion, and c-axis preferred orientation. However, excessively high deposition rates often lead to a kinetic locking effect, where atoms are buried by subsequent particles before they can complete long-range diffusion to lattice sites. This introduces a high density of point defects or dislocations and suppresses the preferential growth of (00*l*) planes. The systematic optimization study by Musri et al. confirmed that for Bi_2_Te_3_ thin films, 75 W is the optimal thereby identifying an optimal sputtering power that balances high crystalline quality, electrical conductivity, and the Seebeck coefficient; further increasing this power degrades the performance [51]. In practical processing, the power is typically maintained between 30 W and 75 W to achieve the best trade-off between deposition rate and thermoelectric performance.

#### 3.1.2. Working Pressure and Particle Transport

**Figure 3:**
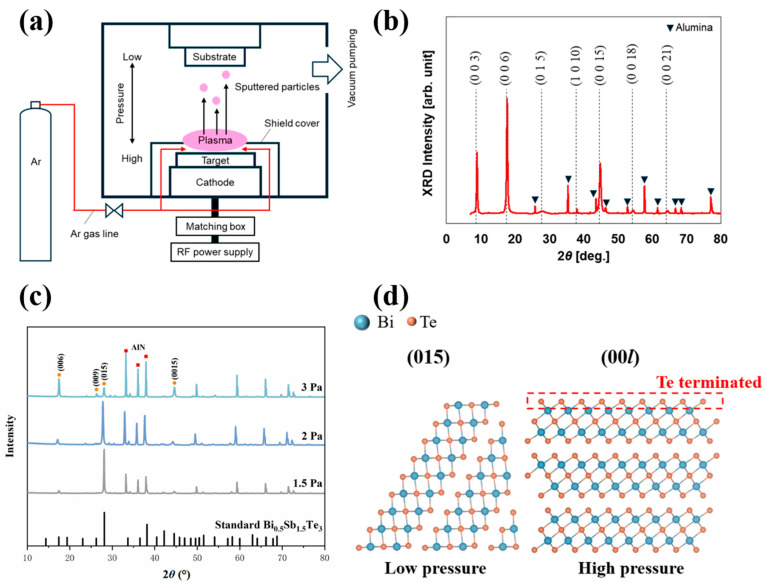
(**a**) Hardware architecture of the PGS system. (**b**) Phase identification and structural analysis of the Bi_2_Te_3_ layer. (**c**) Crystallographic evolution of the Bi_0.5_Sb_1.5_Te_3_ layers deposited across various working pressures. (**d**) Atomistic configurations of the (015) and (00*l*) planes, highlighting the Te-terminated nature of the (00*l*) facet. Note: The Alumina in (**b**) and the AlN in (**c**) are the substrate materials for the thin film (based on [52,53]).

Working pressure is a key physical parameter that regulates the nucleation kinetics and composition transfer of Bi_2_Te_3_ thin films by adjusting the mean free path (MFP) and collision frequency of sputtered particles. At relatively low working pressures, the MFP of sputtered particles is long, and the frequency of collisional scattering among particles is extremely low, allowing most of the initial kinetic energy to be retained until the particles reach the substrate. The impact of such high-energy particles facilitates “congruent transfer” of the target composition and promotes the migration of adatoms on the substrate surface, leading to the formation of flat island structures with a high aspect ratio. Takashiri et al. developed a pressure gradient sputtering (PGS) system (as shown in Figure 3a) and, by exploiting the long MFP characteristics in the low-pressure region between the target and the substrate, successfully fabricated nanocrystalline thin films with an ultrahigh orientation factor [52]. Figure 3b presents the XRD parameters for the thin films that were derived via the following formula [28]:

(1) $$F\left(X\right)=(P-P_{0})/(1-P_{0})$$

(2) $$P=\sum I(X)/\sum I\left(hkl\right)$$

(3) $$P_{0}=\sum I_{0}\left(X\right)/\sum I_{0}(hkl)$$

where *I*(*X*), *I*_0_(*X*), *I*(*hkl*), and *I*_0_(*hkl*) represent the total integrated intensities of the *X* diffraction peak and *P* and *P*_0_ denote the integrated intensity ratios of the (*hkl*) peaks for the oriented and non-oriented samples; and *F* is the orientation factor, reaching a near-perfect value of 0.97. This study demonstrated that the PGS technique can effectively reduce plasma damage while significantly improving the crystalline maintaining thin-film quality even at high deposition rates.

As the working pressure increases, frequent collisions among particles lead to a decay in their average kinetic energy, producing a pronounced “thermalization” effect. However, a high-pressure environment can act as a critical leverage for decoupling transport properties, thereby bolstering the thermoelectric output within defined processing windows. It was revealed in the investigation conducted by Qu et al. that a relatively high deposition pressure (e.g., 3 Pa) effectively suppresses the re-evaporation of tellurium (Te), a volatile element, under thermally activated conditions, thereby maintaining the stoichiometric balance of the system and inducing the formation of a Te-rich (00*l*) surface (Figure 3c) [53]. As shown in Figure 3d, this structural transformation not only shifts the dominant crystallographic orientation from (015) to the thermodynamically more stable (00*l*) direction but also greatly enhances the carrier mobility *μ* by reducing the misorientation angle between grains and the interface barrier *ɸ*_b_. Based on this regulation strategy, a remarkable peak *ZT* of 1.49 was recorded for the Bi_0.5_Sb_1.5_Te_3_ ternary layers at a near-ambient temperature of 313 K, demonstrating the central role of pressure control in optimizing carrier transport efficiency. Furthermore, the working pressure can sensitively tune the microstructure of the thin films by modulating the energy distribution of deposited atoms upon arrival at the substrate.

#### 3.1.3. Substrate Temperature and Crystal Growth

Substrate temperature (*T*_sub_) is a core variable that governs the transition of Bi_2_Te_3_-based thin films from non-equilibrium kinetically controlled growth to thermodynamically equilibrium growth. At low temperatures (below 200 °C), the thermal diffusion capability of adatoms is limited, and their surface diffusion distance remains significantly shorter than the mean spacing between adjacent nuclei. As a result, the film develops a granular, three-dimensional island-like morphology, often accompanied by a high density of point defects and disordered orientations. The kinetic agility of atoms across the substrate surface undergoes a substantial escalation in response to increased thermal input. According to first-principles calculations, the (00*l*) plane of Bi_2_Te_3_ possesses the lowest surface energy (0.16 J m^−2^), much lower than that of the (015) plane (0.37 J m^−2^). Therefore, a high-temperature environment drives atoms to migrate toward the lower-energy (00*l*) sites, promoting the development of a layered structure with a strong c-axis preferred orientation.

Adjusting the thermal pre-treatment from a baseline of 150 °C up to 350 °C yields a dramatic amplification in the film’s charge mobility (from 3.17 to 13.04 cm^2^ V^−1^ s^−1^), as documented by Sakulkalavek et al., the electrical conductivity increased nearly fivefold, and the power factor was also greatly enhanced [36]. Furthermore, Zhu et al. optimized the point defect chemistry within the film through a pronounced “in situ annealing” effect, resulting in denser columnar grains originating from the substrate [54]. The work by Chen et al. further indicated that when *T*_sub_ reaches 450 °C, cross-sectional profiling reveals a pronounced lamellar nanostructure immediately adjacent to the substrate, a structural signature typical of Frank-van der Merwe growth dynamics, whereas at *T*_sub_ = 350 °C, the cross-section exhibits an island-like nanostructure. Consequently, the thermal energy imparted by the substrate acts as the primary thermodynamic driver dictating the crystallographic texture during the onset of film growth.

**Figure 4:**
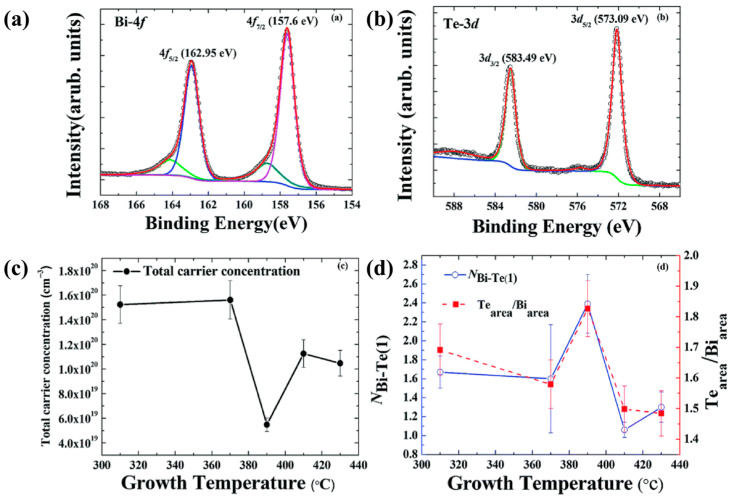
(**a**) Bi 4f and (**b**) Te 3d core-level XPS spectra acquired from the Bi_2_Te_3_ thin film deposited at 390 °C. (**c**) Hall-measured total carrier concentration plotted against growth temperature for Bi_2_Te_3_ films. (**d**) Growth-temperature dependence of the Te/Bi ratio in Bi_2_Te_3_ thin films (based on [55]). Note: The red curve is the fitted curve.

However, the substrate heating temperature itself significantly affects the Bi-to-Te atomic ratio during the sputtering process. Chuang et al. performed XPS measurements on Bi_2_Te_3_ thin films grown at different substrate temperatures [55]. Figure 4a,b show the Bi 4f and Te 3d core-level spectra of a representative film grown at 390 °C. The Te/Bi atomic ratio was calculated using the integrated peak areas. As shown in Figure 4d, as the growth temperature increases from 310 °C to 390 °C, the Te/Bi ratio gradually increases, reaching a maximum at 390 °C. This behavior indicates that within this temperature range, Te atoms progressively diffuse toward the outermost surface. When the growth temperature further rises above 390 °C, the Te/Bi ratio decreases monotonically, which can be attributed to enhanced thermal desorption of Te from the surface. The Te-rich condition at 390 °C corresponds to the minimum electron carrier concentration obtained from Hall measurements (Figure 4c), suggesting that under this growth condition, the concentration of acceptor-like Bi_Te_ antisite defects is maximized, partially compensating the electrons donated by donors (likely Te vacancies). Above 390 °C, the loss of Te suppresses the formation of Bi_Te_ and may favor the generation of donor-type Te_Bi_ antisite defects, leading to a higher electron carrier concentration and a downward shift in the Dirac point relative to the Fermi level. Therefore, XPS provides critical evidence that the growth-temperature-dependent stoichiometry directly influences the antisite defect equilibrium, which in turn tunes the electronic structure of Bi_2_Te_3_ thin films.

#### 3.1.4. Target Composition and Thin Film Stoichiometry

Due to significant differences in the thermophysical properties of tellurium (Te) and bismuth (Bi), the thin film can easily deviate from the ideal stoichiometric ratio of 2:3 during deposition. The primary cause is the difference in their saturated vapor pressures. Investigations conducted by Xu and co-workers revealed that near 1042 K, the vapor pressure of Te is ~10 kPa, while that of Bi is only about 10 Pa [56]. This order-of-magnitude difference makes Te highly prone to re-evaporation during sputtering bombardment and nucleation, so that single-target sputtering typically produces non-stoichiometric thin films with Te deficiency [57]. To precisely compensate for Te loss, the study by Takashiri et al. demonstrated that using a Te-rich target with a special composition (e.g., 28 at.% Bi and 72 at.% Te) is an effective route to bring the film composition toward equilibrium [52]. Furthermore, dual-target simultaneous sputtering from a compound Bi_2_Te_3_ target and a pure tellurium target and a Te target allows for independent adjustment of the power for each target, enabling precise control over the Bi:Te atomic ratio over a wide range from 1.9 to 3.2.

**Figure 5:**
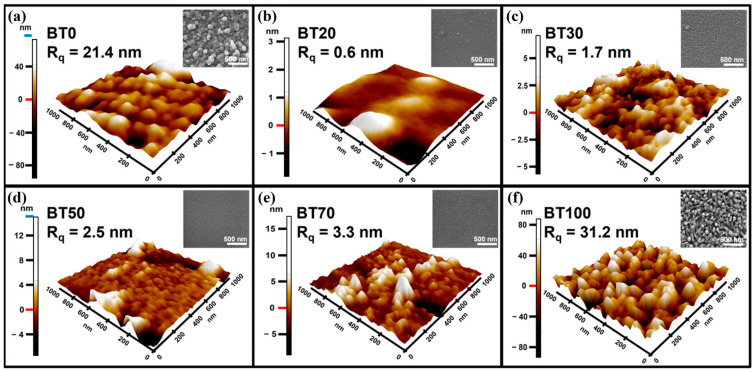
AFM images of thin films prepared under different sputtering parameters, (**a**) BT0, (**b**) BT20, (**c**) BT30, (**d**) BT50, (**e**) BT70, and (**f**) BT100, where the numbers represent the relative atomic percentage of Te in the films (based on [58]).

Following a similar co-sputtering approach with pure elemental targets, Mun et al. effectively controlled the final chemical composition of the deposited films by systematically adjusting the radio frequency (RF) sputtering powers of the bismuth (Bi) target (5–15 W) and the tellurium (Te) target (10–30 W). Fabricating a series of bismuth telluride thin films with varying Te atomic percentages (designated as BT0 to BT100, where the numbers represent the relative atomic percentage of Te) [58]. As illustrated in Figure 5a–f, atomic force microscopy (AFM) characterizations demonstrated that the pristine Bi thin film (BT0) and pristine Te thin film (BT100) exhibited extremely high root-mean-square roughness (*R*_q_) values of 21.4 nm and 31.2 nm, respectively, owing to their large grain sizes. In stark contrast to the pristine thin films, the intermediate-composition thin films (BT20 to BT70) displayed relatively smooth surfaces with no distinct isolated grains observed under the microscope, resulting in substantially reduced *R*_q_ values.

The compositional deviation of the film directly determines its thermoelectric transport characteristics and carrier polarity through the regulation of intrinsic point defect types. Studies have shown that in a Te-rich environment, excess Te atoms tend to occupy Bi sites, forming Te_Bi_ anti-site defects, which act as donors and induce n-type conduction in the film [59]. In contrast, a Te-deficient (Bi-rich) environment promotes Bi atoms to occupy Te sites, forming Bi_Te_ anti-site defects, which produce an acceptor effect and result in p-type characteristics. The experiments by Musri et al. further confirmed that even slight compositional deviations significantly shift the Fermi level position, and excessive deviation from stoichiometry leads to lattice constant contraction and reduced crystallinity, thereby impairing carrier mobility [51]. Therefore, in magnetron sputtering processes, maintaining the Bi:Te atomic ratio slightly above 1.5 through target composition design or co-sputtering compensation is an essential condition for curbing intrinsic anti-site defects while concurrently elevating the electrical power factor [60].

The surface energy, lattice matching, and structural symmetry of the substrate are key factors determining the quality of van der Waals epitaxy (vdW Epitaxy) of Bi_2_Te_3_. Substrates can be broadly classified into two categories: rigid and flexible. On rigid single-crystal substrates with hexagonal symmetry (e.g., MgO or Si (111)), Bi_2_Te_3_ tends to grow with a preferred orientation along the c-axis. Because such substrates provide a good geometric template, the films typically exhibit a clear step-terrace flow growth mode and form flat atomic terraces with large grains, which is crucial for reducing carrier scattering [61]. Flexible polymer substrates are mainly used in wearable thermoelectric applications; poly(ethylene terephthalate) (PET) and polyimide (PI) have emerged as a focal point of recent research, primarily owing to their exceptional mechanical resilience. A pronounced thermomechanical incompatibility exists at the interface, driven by the stark disparity in thermal expansivities between the deposited layer and the non-crystalline base. Data comparison: the CTE of Bi_2_Te_3_ is ~20 × 10^−6^ K^−1^, while that of a typical PI substrate is only 12 × 10^−6^ K^−1^. However, according to the interfacial strain regulation mechanism, despite the physical mismatch, studies have found that by controlling the process to induce a specific island-like growth structure in the film, a microscopic “stress buffer zone” can be created. This structure effectively decouples mechanical strain during macroscopic bending, prevents the formation of through-thickness cracks in the continuous film, and thus significantly improves the adhesion and cyclic fatigue life of flexible devices [62].

### 3.2. Regulation of Microstructure and Properties by Annealing Treatment

#### 3.2.1. Effect of Annealing Temperature on Grain Growth and Defect Elimination

**Figure 6:**
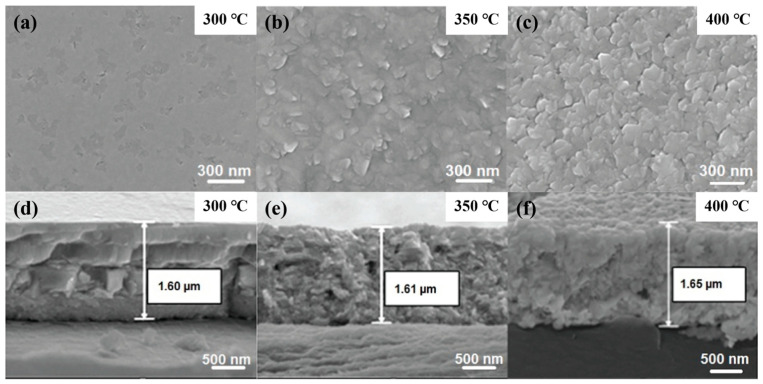
(**a**–**c**) Morphology evolution of Bi_2_Te_3_ thin films under different heat treatment conditions: post-annealing at (**a**) 300 °C, (**b**) 350 °C, and (**c**) 400 °C. (**d**–**f**) Evolution of cross-sectional morphology of Bi_2_Te_3_ thin films under different heat treatment conditions: post-annealing at (**a**) 300 °C, (**b**) 350 °C, and (**c**) 400 °C (based on [50]).

Annealing is a critical post-treatment process for optimizing the thermoelectric performance of bismuth telluride (Bi_2_Te_3_) thin films. Its core function lies in modulating the crystallinity, microstructure, and intrinsic point defect concentration of the films. The study by Singkaselit et al. demonstrates that the annealing temperature directly determines the structural evolution pathway and the final electrical transport properties of the films [50]. In the lower temperature range (e.g., 300 °C), annealing effectively promotes atomic migration and rearrangement, thereby significantly enhancing the crystallinity and increasing the grain size. This grain growth process reduces the number of grain boundaries within the film, thereby weakening carrier scattering at grain boundaries and consequently enhancing the macroscopic carrier mobility. As shown in Figure 6a,d, appropriate thermal treatment helps eliminate residual stress generated during film deposition, resulting in a smoother surface and a denser internal structure. However, when the annealing temperature rises to 350 °C or higher, the defect evolution mechanism of the Bi_2_Te_3_ film changes markedly.

Owing to the high saturated vapor pressure of tellurium (Te), the elevated temperature intensifies the volatilization of Te atoms, generating a large number of Te vacancies (V_Te_) within the film. These defect reactions driven by Te loss not only alter the carrier concentration but can also induce irreversible phase transformation at extremely high temperatures (e.g., above 350 °C), converting the original Bi_2_Te_3_ structure into a Bi-rich BiTe structure. As illustrated in Figure 6b,c,e,f, this phase transformation is accompanied by a decrease in internal density and a deterioration in mechanical hardness, as well as a surge in the free carrier concentration, leading to a reduction in the Seebeck coefficient. Zheng et al. deposited n-type Bi_2_Te_3_ thin films. As the annealing temperature increased from 100 °C to 400 °C, grain growth along the c-axis direction and crystallinity were significantly enhanced, and an optimal power factor of 1.10 × 10^−3^ W m^−1^ K^−2^ was obtained at 300 °C [63]. Raising the annealing temperature from 100 °C to 300 °C led to higher XRD peak intensities, which suggests grain growth and enhanced crystalline quality following the annealing process. However, when the temperature was raised to 400 °C, the intensity of the (015) peak decreased. Thus, overly high annealing temperatures can cause the evaporation of select elements and boost oxygen traces, which consequently raises defect levels and aggravates thermal stress. Therefore, precise control of the annealing temperature is crucial for balancing grain boundary scattering, defect concentration, and phase stability in the thin films.

#### 3.2.2. Effect of Annealing Time on Grain Growth and Defect Elimination

**Figure 7:**
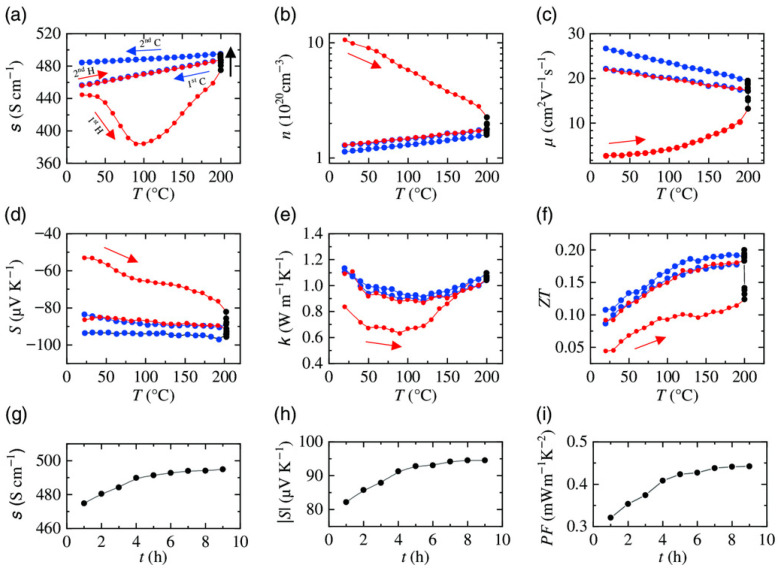
In situ transport characterization of a 90 nm Bi_2_Te_3_ film annealed at 200 ℃ for 9 h. Shown are the heating (red), annealing (black), and cooling (blue) sequences in the temperature range from 20 to 200℃ for two cycles, where first H and first C indicate the first and heating and cooling cycle and second H and second C indicate the second heating and cooling. In the first cycle step, the sample was annealed for 4 h, followed by a second cycle with 5 h annealing time. Temperature-dependent. (**a**–**f**) map the temperature-induced variations in electrical conductivity, charge carrier density, carrier mobility, Seebeck coefficient, overall thermal conductivity, and *ZT*. Panels (**g**–**i**) specifically highlight the kinetic responses of electrical conductivity, Seebeck coefficient, and power factor as a function of the isothermal annealing time (based on [64]).

In contrast to annealing temperature, there have been relatively few systematic investigations into how annealing time affects grain growth in Bi_2_Te_3_ are relatively scarce. Nevertheless, some studies have demonstrated that annealing time is also an important factor influencing grain growth of Bi_2_Te_3_. He et al. prepared n-type Bi_2_Te_3_-based thin films by DC magnetron sputtering at 300 °C and systematically investigated the effect of in situ annealing time on the thermoelectric properties of the films. The results showed that increasing the sputtering power led to a higher deposition rate and larger grain size. After determining the optimal sputtering power of 30 W, the performance was further optimized through an in situ annealing process. The results indicated that the film annealed for 40 min exhibited a significant decrease in electrical conductivity due to a reduction in carrier concentration, while the Seebeck coefficient was enhanced. A maximum power factor of 0.82 mW m^−1^ K^−2^ was obtained at 543 K [54]. This finding suggests that an appropriate annealing time is crucial for balancing carrier concentration and the Seebeck coefficient, thereby optimizing thermoelectric performance. Naumochkin et al. systematically studied the impact of annealing on sputtered Bi_2_Te_3_ thin films, focusing on the roles of annealing duration and temperature in 90 nm-thick samples. Experiments illustrated in Figure 7a–i show that film thickness and optimal annealing time are linked—namely, thicker films demand extended annealing durations to attain peak *ZT* and power factor [64]. This finding reveals a coupling effect between annealing time and material scale, providing valuable guidance for the design of annealing processes for films of different thicknesses in practical applications.

#### 3.2.3. Role of Annealing Atmosphere (Vacuum, Inert, Reducing)

The effects of annealing temperature and time on grain growth and defect elimination in Bi_2_Te_3_ have been systematically discussed above. However, the microstructure and thermoelectric behavior of Bi_2_Te_3_ are also strongly influenced by the annealing atmosphere. Since Bi_2_Te_3_ contains the volatile element Te, the annealing atmosphere directly affects the volatilization behavior of elements, defect evolution, and phase stability, consequently setting the materials’ electrical and thermal conduction behavior. According to the mechanisms of different atmospheres, three types of annealing can be classified: vacuum annealing, inert atmosphere annealing, and reducing atmosphere annealing.

**Figure 8:**
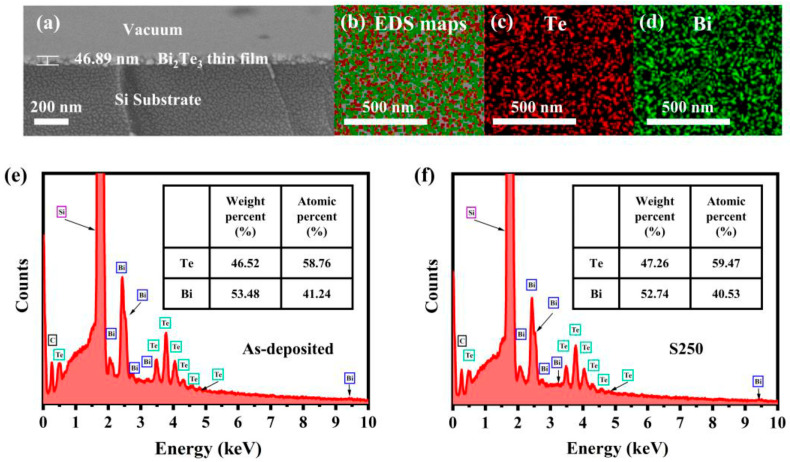
The cross-sectional SEM image of (**a**) the S250 sample; (**b**–**d**) element mapping images of the S250 sample for Te (red dots) and Bi (green dots); EDS spectrums of (**e**) the as-deposited and (**f**) the S250 sample. Note: In (**b**), the pink part represents the element C (based on [65]).

Scanning electron microscopy (SEM) was employed to characterize the cross-sectional morphology of the thin films after annealing. As illustrated in Figure 8a, the film thickness of the S250 sample (annealed at 250 °C) is estimated to be ~47 nm, where the vacuum environment, Bi_2_Te_3_ layer, and Si substrate are clearly distinguished, demonstrating excellent film formation. Vacuum annealing is a critical heat treatment method for Bi_2_Te_3_ materials, as its core function is to regulate the elemental stoichiometry and structural evolution of the films. Zhang et al. systematically investigated the compositional optimization and defect elimination of sputtered Bi_2_Te_3_ thin films during vacuum thermal processing [65]. As shown in the EDS mapping of Figure 8b–d, the Bi and Te elements are uniformly and continuously distributed across the substrate after annealing, indicating high film quality without noticeable elemental segregation. Figure 8e,f reveal that for the as-deposited specimen, the atomic ratio of Bi to Te is ~0.70, representing a notable deviation from the ideal stoichiometry; this indicates that the initial deposition process introduces a certain degree of elemental disorder. A clear correlation exists between vacuum annealing and compositional stabilization. Specifically, after annealing at 250 °C, the atomic ratio of Bi to Te decreases to 0.68, which is significantly closer to the standard stoichiometric ratio of 0.67 for Bi_2_Te_3_. The effective thermal activation promotes atomic rearrangement from a disordered amorphous state into a highly ordered crystalline structure. This microstructural evolution significantly reduces internal defects such as grain boundaries, vacancies, and interstitial atoms, thereby fundamentally enhancing the physical and thermoelectric properties of the sample. This research demonstrates that appropriate vacuum annealing effectively minimizes stoichiometric deviations and structural defects, revealing the underlying mechanism of composition homogenization and quality improvement during thermal treatment.

Inert atmosphere annealing (e.g., Ar or N_2_ atmosphere) primarily optimizes thermoelectric performance by suppressing oxidation and regulating the microstructure. Compared to vacuum annealing, an inert atmosphere can inhibit excessive volatilization of Te to a certain extent while providing a relatively stable thermal environment. Wang et al. used N_2_ as a shielding gas during annealing and obtained a stable Bi_2_Te_3_/W multilayer structure. The multilayer structure of Bi_2_Te_3_/W thin films can reasonably regulate the carrier concentration and filter low-energy carriers, thereby simultaneously enhancing the electrical conductivity and Seebeck coefficient. The power factor of Bi_2_Te_3_/W reaches 1785 μW m^−1^ K^−2^ at 600 K [66]. Compared to vacuum annealing, which is prone to severe Te volatilization, and inert gas annealing, which only passively prevents oxidation, reducing atmosphere annealing (such as a hydrogen-argon mixture) can actively and chemically reduce the insulating oxides (e.g., Bi_2_O_3_ and TeO_2_) on the film surfaces and grain boundaries into pure metallic elements. This process deeply purifies the grain boundaries and interfacial defects of the material without causing severe stoichiometric deviations. Consequently, reducing atmosphere annealing substantially weakens the interfacial scattering of charge carriers caused by insulating barriers, thereby significantly enhancing the carrier mobility, electrical conductivity, and the overall power factor of bismuth telluride thin films [67].

#### 3.2.4. Comparison Between Rapid Thermal Annealing and Conventional Tube Furnace Annealing

The core difference between different annealing methods lies in the temperature and time history of the heat treatment, which directly determines the evolution path of the material’s microstructure. Specifically, conventional tube furnace annealing (TFA) typically employs a slow heating rate (5–10 °C min^−1^) and a long holding time (several hours or even tens of hours), relying on thermal radiation and convection heating [65,68]. In contrast, rapid thermal annealing (RTA) uses radiative heating sources such as lasers, allowing the heat treatment process to be completed within seconds to minutes. The production rate of Bi_2_Te_3_ was around 180 times higher than that of conventional thermal annealing, thereby significantly shortening the process cycle [69]. From an equipment perspective, rapid thermal annealing furnaces differ significantly from conventional tube furnaces in terms of heating method, heating efficiency, temperature range, and structural design.

In recent years, rapid thermal annealing has achieved notable progress in the research of Bi_2_Te_3_ thin-film thermoelectric materials, attracting widespread attention due to its short-duration, high-efficiency characteristics. Zheng et al. prepared Bi_2_Te_3_ nanocrystalline thin films on polymer substrates using a single-source evaporation method and applied rapid thermal treatment. The study found that rapid annealing effectively improved the crystallinity of the films and enabled control over grain size, yielding a pure Bi_2_Te_3_ phase with nanoscale grains and ultimately increasing the *ZT* value to 0.5 [70]. N-type and p-type Bi_2_Te_3_ thin films were fabricated by Rashid et al. via constant-current electrodeposition, followed by a systematic study of the RTA treatment’s impact on the microstructural and thermoelectric characteristics. The Seebeck coefficient of the n-type thin film increased from −57 μV K^−1^ to −169 μV K^−1^, and that of the p-type film increased from 28 μV K^−1^ to 112 μV K^−1^; the power factor was enhanced approximately sixfold for the n-type film and about twofold for the p-type film [71]. This research clearly shows that a short-duration rapid thermal treatment can greatly boost the thermoelectric properties of Bi_2_Te_3_, highlighting distinct advantages in terms of the process.

Conventional tube furnace annealing, as a standard method for material heat treatment, is also widely used in the research of Bi_2_Te_3_-based materials. Yamashita and Tomiyoshi reported that after vacuum annealing of p-type Bi_2_Te_3_ at 673 K for 2 h, the room-temperature power factor increased from 4.25 × 10^−3^ W m^−1^ K^−2^ to 5.72 × 10^−3^ W m^−1^ K^−2^, representing an improvement of ~35% [72]. However, conventional tube furnace annealing typically requires several hours or even longer processing times, resulting in a long process cycle and high energy consumption. Moreover, prolonged high-temperature heat treatment may induce volatilization loss of Te and compositional deviation.

### 3.3. Research Progress on Doping Modification

#### 3.3.1. n-Type Doping

To achieve n-type doping, incorporating selenium to create Bi_2_Te_3-*x*_Se*_x_* solid solutions proves effective for boosting thermoelectric performance. Bi_2_Te_3_ and Bi_2_Se_3_ share the same rhombohedral crystal structure, and they can form a continuous solid solution Bi_2_Te_3-*x*_Se*_x_* over the entire composition range [73]. During the formation of the solid solution, Se atoms with higher electronegativity preferentially occupy Te lattice sites. Because selenium has a smaller atomic radius than tellurium, the lattice constant steadily decreases as the Se doping level rises. Meanwhile, the formation of Bi-Se bonds enhances the chemical bonding strength within the lattice, leading to a gradual increase in the band gap [74].

In Bi_2_Se_3_, the formation energies of the anti-site defects Bi_Te_ and Te_Bi_ are similar (~0.6 eV), allowing both to coexist at relatively high concentrations and thereby inducing a high density of diverse microstructures (e.g., ripple dislocations, edge dislocations, staggered bilayer structures, etc.). In contrast, in Bi_2_Se_3_, the formation energies of the anti-site defects Bi_Se_ and Se_Bi_ are relatively high (~1.3 eV), making it difficult for both to form simultaneously in large quantities, which results in a more regular atomic arrangement [75]. Consequently, when Se is incorporated into the Bi_2_Te_3_ lattice, the defect characteristics and microstructure of the system change, thereby affecting the mechanical properties and thermoelectric transport behavior. The results indicate that when the Se solid solution content is below 20%, Bi_2_(Te, Se)_3_ crystals still maintain good plastic deformability (maximum bending strain ≥ 10%) while simultaneously exhibiting high thermoelectric performance.

As the Se doping content increases, the carrier concentration of the system exhibits a decreasing trend, which is mainly attributed to the modulation of defect equilibrium and band structure by Se doping. The decrease in carrier concentration leads to a reduction in electrical conductivity on the one hand, but on the other hand significantly enhances the Seebeck coefficient, which is attributed to the effective increase in the density of states and the shift in the Fermi level toward the interior of the band gap. This research has shown that by optimizing the Se doping concentration, an optimal trade-off between electrical conductivity and the Seebeck coefficient can be achieved, thereby maximizing the power factor [76].

#### 3.3.2. p-Type Doping

Unlike the Se substitution at Te sites in n-type Bi_2_Te_3-*x*_Se*_x_* solid solutions, the p-type doping strategy mainly employs Sb substitution at Bi sites to form (Bi_1-*x*_Sb*_x_*)_2_Te_3_ solid solutions. Sb and Bi belong to the same group of VA elements and possess similar chemical properties and electronic configurations; therefore, Sb substitution for Bi can effectively convert the conduction type from n-type to p-type while maintaining the integrity of the crystal structure [77,78]. Bi_2_Te_3_ crystallize in the rhombohedral structure (space group R3m) and can mix to form a continuous solid solution over a wide range of compositions. The atomic size of Sb (~1.59 Å) is marginally smaller than that of Bi (~1.63 Å). Consequently, higher Sb substitution levels lead to a monotonic drop in the lattice constant, analogous to the contraction effect caused by Se doping in n-type systems [75].

**Figure 9:**
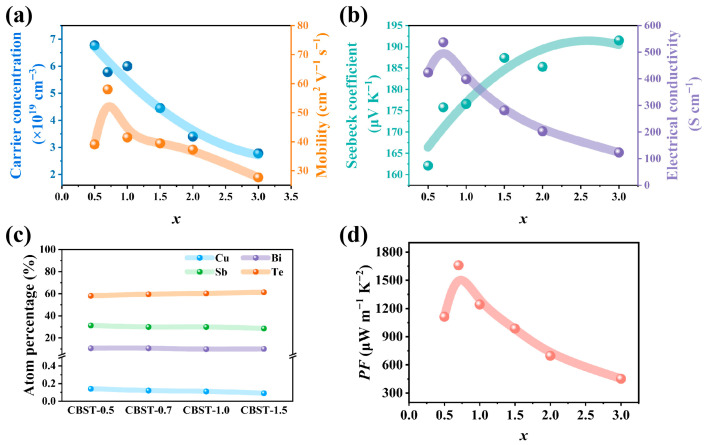
Variation of (**a**) *n* and *μ*, (**b**) *S* and *σ* of CBST-*x* (*x* = 0.5, 0.7, 1.0, 1.5) thin films. (**c**) EDS detected atomic percentages of CBST films prepared at different working pressures (0.5–1.5 Pa). (**d**) Variation in *PF* of CBST-*x* (*x* = 0.5, 0.7, 1.0, 1.5) thin films (based on [79]).

Building upon this fundamental (Bi,Sb)_2_Te_3_ (BST) solid solution framework, further doping modification and stoichiometric regulation serve as cornerstone strategies for fine-tuning carrier transport properties and suppressing the detrimental bipolar effect. Specifically, the incorporation of transition metal elements like copper (Cu) into the p-type BST system has demonstrated remarkable efficacy in performance optimization. Cu is particularly favored as an external dopant because its electronegativity and atomic radius are comparable to those of Sb. During the magnetron sputtering process, Cu atoms preferentially occupy Sb sites in the lattice, functioning as acceptor dopants that introduce additional holes into the system. This mechanism effectively elevates the hole carrier concentration and compensates for minority electron generation, thereby suppressing the intrinsic excitation that typically degrades thermoelectric performance at elevated temperatures. Research by Ge et al. shows that optimized Cu-doped films to exhibit an optimized carrier concentration of 5.78 × 10^19^ cm^−3^ and an enhanced mobility of 58.0 cm^2^ V^−1^ s^−1^ at room temperature, while achieving a peak Seebeck coefficient of 203 μV K^−1^ at 400 K due to the effective mitigation of the bipolar effect (Figure 9a,b) [79].

As shown in Figure 9c, beyond external element doping, “self-doping” via stoichiometric adjustment remains a critical regulatory dimension. The intrinsic carrier concentration in BST systems is primarily governed by the dynamic equilibrium between antisite defects (e.g., Sb_Te_, Bi_Te_) and tellurium vacancies (V_Te_). During sputtering, process parameters such as working pressure significantly influence the “re-sputtering” effect of weakly bonded Sb atoms, thereby shifting the Te/(Bi + Sb) ratio and altering the concentration of hole-generating antisite defects.

Recently, these synergistic approaches have challenged the traditional paradigm that high thermoelectric performance is exclusively tied to the (00*l*) preferential orientation. By coupling optimized Cu-acceptor doping with precise pressure-controlled defect and grain growth engineering, non-(00*l*) layered flexible p-type films (e.g., (015) oriented) have achieved a room-temperature power factor (*PF*) of 1660 μW m^−1^ K^−2^ (Figure 9d). This represents one of the highest reported values for non-(00*l*) oriented films, underscoring the potential of combining structural engineering with multi-element doping to develop high-efficiency, flexible thermoelectric devices. To provide a comprehensive comparison, the thermoelectric performance parameters of various doped bismuth telluride thin films are summarized in Table 1.

## 4. Conclusions and Outlook

Recent milestones achieved in the magnetron sputtering deposition of Bi_2_Te_3_-based layers are comprehensively outlined in this article, with an emphasis on the intrinsic relationships among process parameters, microstructures, and thermoelectric performance. Ultimately, the microstructural evolution and growth kinetics of the thin films are governed by a synergistic interplay of deposition variables—primarily discharge power, chamber pressure, substrate temperature, and source stoichiometry. These factors, along with the substrate type, critically determine the film growth mode (island, layered, or mixed), stoichiometry, defect chemistry, and preferred orientation, thereby dictating the resulting Seebeck coefficient, electrical conductivity, and thermal conductivity. Post-annealing treatments—including temperature, time, atmosphere (vacuum, inert, or reducing), and method (conventional tube furnace versus rapid thermal annealing)—effectively modulate grain size, crystallinity, point defects, and Te volatilization, effectively optimizing the trade-off between electrical and thermal transport, leading to a concomitant boost in both power factor and *ZT* value. Doping modification, particularly n-type Se doping and p-type Sb doping, adjusts the carrier concentration and band structure while also altering defect formation energies and microstructural features (e.g., dislocations and layered defects), offering additional degrees of freedom for optimizing both thermoelectric and mechanical properties. Despite these advances, several challenges remain. The lack of systematic comparability across studies due to widely varying deposition conditions hinders the establishment of general process—structure—property maps. Furthermore, the scalability of magnetron sputtering for industrial production of flexible thermoelectric devices requires better control over large-area uniformity, substrate compatibility, and long-term stability. Future research should focus on establishing high-throughput experimental or machine-learning-assisted frameworks to rapidly identify optimal sputtering conditions, exploring novel multilayer or superlattice architectures to further decouple electrical and thermal transport, developing in situ characterization techniques to monitor growth and annealing dynamics, and integrating Bi_2_Te_3_ thin films into functional microdevices (e.g., on-chip coolers and wearable sensors) with demonstrated reliability. Addressing these issues will accelerate the practical application of magnetron-sputtered Bi_2_Te_3_ thin films in next-generation energy harvesting and thermal management systems.

## Figures and Tables

**Table 1:** Comparison of thermoelectric performance parameters of Bi_2_Te_3_-based thin films after different doping.

Materials	Conductivity Type	*σ* (S·cm^−1^)	*n* (10^20^ cm^3^)	*μ* (cm^2^ V^−1^ s^−1)^	*S* (μV K^−1^)	*PF* (μW cm^−1^ K^−2^)	Ref.
Cu-doped Bi_0.5_Sb_1.5_Te_3_	p type	537	0.578	58.0	203	16.6	[79]
Ti-doped Bi_2_Te_3_	n type	1900	4.23	28	−187	66.6	[80]
W-doped Bi_0.5_Sb_1.5_Te_3_	p type	~280	~0.13	~120	~225	~13.2	[81]
S-doped Bi_2_Te_3_	n type	1477	-	-	−93	14.8	[82]
Ag-doped Bi_0.5_Sb_1.5_Te_3_	p type	122	0.90	50.8	129	12.4	[83]
Bi_2_Te_3_Se_0.3_	n type	730	0.39	114	−182	23	[84]
Bi_0.5_Sb_1.5_Te_3_	p type	493	-	-	154	11.7	[85]

## Data Availability

No new data were created or analyzed in this study. Data sharing is not applicable to this article.
